# The prevalence and behavioral risk factors contributing to non-communicable diseases in Bushbuckridge, Mpumalanga province, South Africa

**DOI:** 10.3389/fepid.2025.1560971

**Published:** 2025-04-10

**Authors:** Thabo D. Pilusa, Cairo B. Ntimana, Eric Maimela

**Affiliations:** ^1^ Department of Public Health, University of Limpopo, Polokwane, South Africa; ^2^ DIMAMO Population Health Research Centre, University of Limpopo, Polokwane, South Africa

**Keywords:** physical inactivity, alcohol consumption, health care workers, hypertension, non-communicable disease, behavioural risk factors

## Abstract

**Background:**

Intervention strategies such as health campaigns, pre-screening, health education, and health talks exist. Still, they are only active if there are outbreaks of the specific infectious disease not mainly NCDs. Therefore, there is a need to develop intervention strategies to improve the prevention and control of behavioral risk factors for NCDs by determining social, economic, and health system factors. Hence, the study aimed to determine the prevalence and determinants of behavioral risk factors contributing to NCDs in Bushbuckridge, South Africa.

**Methods:**

This cross-sectional descriptive study involved 2,400 respondents selected from healthcare facilities. The participants were selected using simple random sampling. Data was analyzed using SPSS version 29. A comparison of proportions was performed using the chi-square test. The association between sociodemographic and lifestyle factors with predictors of behavioral risk factors for NCD was analyzed using binary regression analysis, and the statistical significance was set at a *p*-value of <0.05.

**Results:**

The mean age of the study was 46.27 ± 13.38. The prevalence of Smoking was 51.3% (1,211). The prevalence of alcohol consumption within the past year was 19.3% (463), while inadequate fruit and vegetable intake was 76.2%. Physical inactivity was 97.2%. Additionally, hypertension and diabetes were 51% and 50.1% respectively. Participants (≥35 years) were likely to have low fruit and vegetable intake (aOR = 1.3; 95% CI: 0.99–1.62). Widows were 30% less likely to smoke (aOR = 0.72; 95% CI: 0.57–0.92), yet they were 1.4 times more likely to consume alcohol (aOR = 1.4; 95% CI: 0.99–1.84). Unemployed participants were found to have a higher likelihood of consuming alcohol (aOR = 1.3; 95% CI: 1.02–1.54).

**Conclusion:**

The prevalence of behavioral risk factors for NCDs was found to be high among rural populations residing in Bushbuckridge, underscoring the need for sustained and comprehensive interventions. In rural areas like Bushbuckridge, the combination of poverty, unemployment, limited healthcare access, and evolving social dynamics creates a challenging environment that fosters unhealthy behaviors and increases the risk of NCDs. To effectively reduce the burden of these diseases in such communities, public health strategies must focus on socio-economic and cultural determinants, rather than just demographic factors.

## 1 Introduction

Non-communicable diseases (NCDs), tend to be long duration and require lifelong medication which occurs as the result of a combination of genetic, physiological, environmental, and behavioral factors (1, 2). The main types of NCDs are heart attacks, hypertension, diabetes, stroke, cancers, chronic respiratory diseases, and asthma (3). The threat of non-communicable diseases (NCDs) is growing globally. Unfortunately, there are significant obstacles in the fight against these challenges (4–6). NCDs are a serious public health threat because they affect the current population and may also burden future generations if preventive measures are not taken to reduce their prevalence and impact (4). The increasing prevalence of NCDs causes a negative impact on health services as these conditions include a wide range of devastating conditions such as type 2 diabetes, cardiovascular diseases, cancers, chronic obstructive airway diseases, and mental health disorders (4). Since these conditions require ongoing treatment, South Africa is one of the countries facing excessive expenditure in managing the high burden of NCDs (4). According to Owalade et al. (5), NCDs are increasing health problems globally, they have overtaken conditions such as undernourishment, marasmus, HIV, and tuberculosis. Globally, 15 million people aged 30–69 years die prematurely each year due to non-communicable diseases (NCDs), and the economic burden of these diseases is projected to reach USD 47 trillion over the coming decades (7). This burden adds to the already overburdened health care service in South Africa due to the need to manage the already existing problem of multi-morbidity of HIV, Mycobacterium TB, and Diabetes mellitus (4).

The four main behavioral risk factors for NCDs are tobacco use, unhealthy diet, physical inactivity, and harmful use of alcohol (3, 8, 9) which contribute to 80% of the NCD burden globally (4). Despite significant heterogeneity in exposure and outcome measures, clear evidence shows that the burden of behavioural risk factors is affected by socioeconomic position within low-middle-income countries (10). Governments seeking to meet Sustainable Development Goal (SDG) 3 reducing premature non-communicable disease mortality by a third by 2030 should leverage their development budgets to address the poverty-health nexus in these settings (10).

The hazardous effects of behavioral and dietary risk factors on NCDs, and the metabolic and physiological conditions that mediate their effects, have been established in prospective cohort studies and randomized trials (11–13). This knowledge, together with data from risk-factor surveillance of physical inactivities, unhealthy diet, tobacco use, and alcohol consumption has helped to establish the mortality and disease burden attributable to risk factors, globally and by region and country (11, 14). Studies show that the vast majority of NCDs can be prevented through behavioral risk-reduction interventions (15, 16). Properly executed, the interventions could lead to a decrease in the burden of NCDs, ranging from a 30% drop in the prevalence of cancer to a 75% reduction in cardiovascular diseases (17). The impact of NCDs in African countries already burdened with communicable diseases ranges from losses in economic productivity to the diversion of resources toward managing these conditions. South Africa is no exception (16). In Mpumalanga, Enhlazeni, the following intervention strategies such as health campaigns, pre-screening, health education, and health talks exist but they are only active if there are outbreaks of the specific infectious disease not mainly NCDs. Therefore, there is a need to develop intervention strategies to improve the prevention and control of behavioural risk factors for NCDs by determining social, economic, and health system factors. This is mainly because NCDs are of increasing concern for society and national governments, as well as globally due to their high mortality rate. Thus, the study aimed to determine the prevalence and determinants of behavioral risk factors contributing to NCDs in Bushbuckridge, Ehlanzeni District Municipality of Mpumalanga Province.

## 2 Materials and methods

### 2.1 Design, setting, and participants

This cross-sectional descriptive study involved 2,400 respondents selected from healthcare facilities within the Bushbuckridge Sub-district of Mpumalanga Province, an area characterized by low socio-economic status and predominantly inhabited by individuals of Tsonga origin.

### 2.2 Sampling process

**Figure 1:**
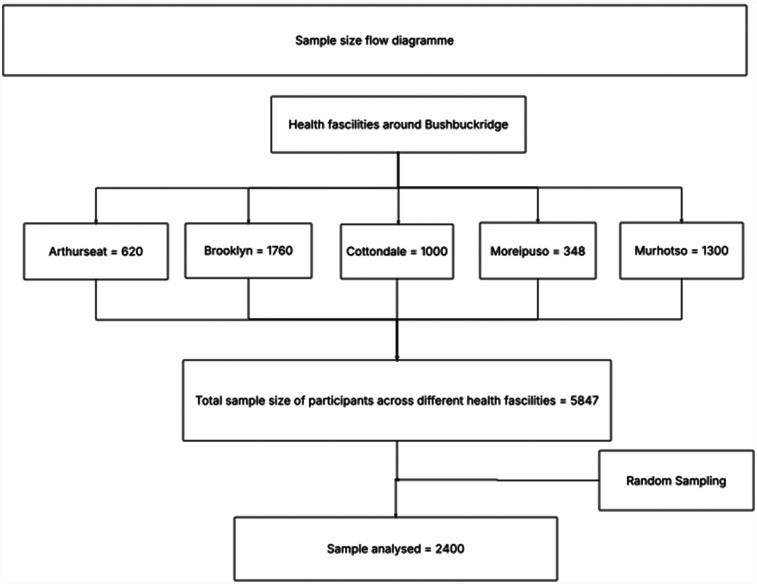
Participants' selection.

The study focused on patients diagnosed with hypertension and/or diabetes, healthcare workers, and community health workers who were knowledgeable about chronic management across six different healthcare facilities in Bushbuckridge, located in the Ehlanzeni District Municipality. The distribution of participants across the facilities was as follows: Arthurseat (620), Brooklyn (1,760), Buffelsoek (819), Cottondale (1,000), Moreipuso (348), and Murhotso (1,300), totaling 5,847 participants. Based on this population size, an estimated sample size of 400 participants was determined. From across each health facility, 400 participants were randomly selected and analyzed. The study, included participants aged 18 and above, and those who are not mentally stable were excluded in the study (Figure 1).

### 2.3 Data collection

The researchers adopted and adjusted the WHO step-wise data collection tool which is an internationally comparable, standardized, and integrated surveillance tool through which countries can collect, analyze, and disseminate core information on non-communicable diseases (18). The questionnaire had questions related to demographics, alcohol consumption, smoking, physical activity, fruit and vegetable consumption, raised blood pressure and, raised blood glucose. (See Supplementary Material S1). Participants who agreed to participate in this study completed and signed a consent form before participating in the study. The data collection tool was presented to expect to be modified according to their inputs. The researcher submitted the tools to the research panel of experts for assessment. The pilot study is a mini-research conducted before the major study (19). The instrument was then adjusted and modified depending on the findings of the pilot study. Piloting the instrument allowed the researchers to check its clarity, readability, feasibility, and suitability for the research.

### 2.4 Data analysis

Data was analyzed using Statistical Package for Social Sciences (SPSS) version 29. Continuous variables that were normally distributed were presented in terms of mean ± standard, categorical variables were presented in terms of percentage, and chi-square test and student t-test were used for comparing groups for categorical and continuous variables respectively. Kolmogorov–Smirnov was used to test the normality of continuous variables. Multivariate logistic regression was used to determine predictors of behavioral risk factors for NCDs. In this analysis, hypertension, diabetes, smoking, alcohol consumption, low fruit and vegetable intake, and physical inactivity were the dependent variables, while sociodemographic profiles (age groups, marital status, employment status, gender, and educational status) served as the independent variables. A *p*-value of less than 0.05 was considered statistically significant.

### 2.5 Ethical consideration

Ethical clearance (Project number: TREC/1777/2023:PG) obtained from the University of Limpopo Turfloop Campus and Mpumalanga Provincial Health Research and Ethics Committee (MPHREC) Reference Number: MP 202403 001. Before the study started, consent forms were given to the participants, who signed them to express their willingness to take part. They were presented with the aim of the study beforehand and made aware of their freedom to withdraw from participation at any moment. Participants received guarantees of anonymity and secrecy, and their rather than their actual names were used to identify them. A password secured the PC that was used to transcribe the interviews.

## 3 Results

The study found that the overall mean age of the participants was 46.27 ± 13.38 years and the mean ages of females and males were 46.71 ± 13.38 years and 45.37 ± 13.33 years respectively. Sixty-seven percent (1,623) of the participants were females and most of the participants were never married [31.5% (756)] females dominated the unmarried participants at 32.7% (254) as compared to males at 30.9% (502) but there was no statistically significant difference between them. The proportion of smoking and alcohol consumption was 50.6% (1,211) and 51.9% (1,245) respectively with no significant association between males and females (Table 1).

**Table 1:** Characteristics of study participants by gender.

Variable	Females (*n* = 1,623)	Males (*n* = 777)	Total (*n* = 2,400)	*P*-value
Educational Status				0.131
No education	306 (18.9%)	147 (18.9%)	453 (18.9%)	
Primary	433 (26.7%)	238 (30.6%)	671 (28.0%)	
Secondary	551 (34.0%)	232 (29.9%)	783 (32.6%)	
Tertiary	333 (20.5%)	160 (20.6%)	493 (20.5%)	
Work Status				0.199
Working	547 (33.7%)	401 (51.6%)	948 (39.5%)	
Not working	1,076 (66.3%)	376 (48.4%)	1,452 (60.5%)	
Marital Status				0.850
Never married	254 (32.7%)	502 (30.9%)	756 (31.5%)	
Married	189 (24.3%)	411 (24.3%)	600 (24.3%)	
Divorced	185 (23.8%)	394 (24.3%)	579 (24.1%)	
Widowed	149 (19.2%)	316 (19.5%)	465 (19.4%)	
Smoking				0.990
Yes	378 (32.4%)	833 (67.6%)	1,211 (50.5%)	
No	790 (67.6%)	399 (32.4%)	1,189 (49.5%)	
Alcohol Consumption				0.866
Yes	840 (51.8%)	405 (52.1%)	1,245 (51.9%)	
No	783 (48.2%)	372 (47.9%)	1,155 (48.1%)	

Table 2, shows that a substantial proportion of both genders do not meet the recommended intake of at least 5 servings per day. Specifically, 76.2% (1,732) of the total participants consume less than 5 servings daily, with males at 75.8% (95% CI: 72.7%–78.7%) and females at 76.4% (95% CI: 74.3%–78.4%). The *p*-value of 0.748 indicates no statistically significant difference between males and females in their consumption patterns.

**Table 2:** Prevalence of low fruit and vegetable intake stratified by gender.

Risk factors	Total	Males (*n* = 777)	Females (*n* = 1,623)	*P*-value for trend (males vs. females)
	% (95% CI)	% (95% CI)	% (95% CI)	
Fruit and vegetable				
<5 servings/day	76.2 (74.5–77.9)	75.8 (72.7–78.7)	76.4 (74.3–78.4)	0.748

Table 3 Presents smoking prevalence data for both current and daily smokers across different age groups for females and males. Among females, current smoking rates show a slight increase with age, peaking at 57.4% (95% CI: 43.9–69.8) in those aged 66 and above (*p* = 0.050). In terms of daily smokers, the prevalence is highest in the 25–34 age group (27%, 95% CI: 19.5%–36.0%) and decreases steadily with age, reaching 9.3% (95% CI: 3.9%–20.4%) in those aged 66 and above, (*p* < 0.001). For males, current smoking rates remain relatively stable across all age groups, ranging from 50% in the 18–24 age group to 49.3% (95% CI: 41.2–57.4) in those aged 66 and above, with no significant difference (*p* = 0.635). The prevalence of daily smoking also decreases with age, from 24.4% (95% CI: 19.3%–30.2%) in the 25–34 age group to 11.8% (95% CI: 7.5%–18.2%) in those aged 66 and above (*p* < 0.001). Risk factors associated with smoking were boredom at 14.3%, peer pressure at 12.3%, family members at 12.2%, and 61.3% of participants who just started smoking out of nothing.

**Table 3:** Prevalence of smoking stratified by sex and age group.

Females (*n* = 1,623)		Age groups						
Risk factor		18–24 years	25–34 years	35–44 years	45–54 years	55–64 years	≥66 years	*P*-value
		% (95% CI)	% (95% CI)	% (95% CI)	% (95% CI)	% (95% CI)	% (95% CI)	
Smoking	Current smokers	42.9	52.3	52.6	50.0	51.9	57.4	0.050
		(30.6–56.0)	(42.9–61.4)	(45.5–59.6)	(43.2–56.8)	(44.2–59.5)	(43.9–69.8)	
	Daily smokers	23.2	27.0	17.4	12.2	15.4	9.3	<0.001
		(13.9–36.0)	(19.5–36.0)	(12.6–23.4)	(9.2–18.6)	(10.6–21.9)	(3.9–20.4)	
Males (*n* = 777)		Age groups						
Risk factor		18–24 years	25–34 years	35–44 years	45–54 years	55–64 years	≥66 years	*P*-value
		% (95% CI)	% (95% CI)	% (95% CI)	% (95% CI)	% (95% CI)	% (95% CI)	
Smoking	Current smokers	50.0	56.7	49.7	50.8	51.1	49.3	0.635
		(39.5–60.5)	(50.3–62.9)	(44.6–54.9)	(46.1–55.5)	(45.9–56.3)	(41.2–57.4)	
	Daily smokers	20.2	24.4	13.1	16.8	19.3	11.8	<0.001
		(12.9–30.2)	(19.3–30.2)	(9.9–16.9)	(13.6–20.6)	(15.6–23.7)	(7.5–18.2)	

Table 4 Overall, 19.3% (95% CI: 17.8–20.9) of participants reported consuming alcohol in the last 12 months, with no significant difference between males and females (*p* = 0.806). In the past 30 days, 13.8% (95% CI: 12.5–15.2) consumed alcohol rarely with meals, 12.6% (95% CI: 11.4–14.0) sometimes with meals, and 12.7% (95% CI: 11.4–14.1) usually with meals (*p* = 0.203).

**Table 4:** Prevalence of alcohol consumption stratified by gender.

Alcohol consumption	Total	Males (*n* = 777)	Females (*n* = 1,623)	*P*-value for trend
	% (95% CI)	% (95% CI)	% (95% CI)	
Consume the last 12 months	19.3 (17.8–20.9)	19.0 (16.4–21.9)	19.5 (17.6–21.5)	0.806
Alcohol in 30 days				
Rarely with meals	13.8 (12.5–15.2)	12.9 (10.8–15.6)	14.2 (12.6–15.9)	0.203
Sometimes with meals	12.6 (11.4–14.0)	14.7 (12.4–17.3)	11.6 (10.2–13.3)	
Usually with meals	12.7 (11.4–14.1)	12.4 (10.2–14.9)	12.9 (11.3–14.6)	

Daily consumers: Males consume alcohol at 10.8% (95% CI: 8.8–11.7) as compared to females at 10.2% (95% CI: 8.8–11.7) daily with no statistical significance (*p* = 0.940) (Table 5).

**Table 5:** The frequency of having at least one alcoholic drink during the past 12 months.

Frequency	Females	Males	*P*-value for trend
1 =< once a month	10.2 (8.8–11.7)	9.9 (7.9–12.2)	0.940
2 = 1–4 days	10.7 (9.3–12.3)	10.2 (8.2–12.5)	
3 = 5–6 days	9.6 (8.3–11.1)	10.4 (8.5–12.8)	
4 daily	10.2 (8.8–11.7)	10.8 (8.8–13.2)	

The prevalence of low physical activity was 97.2% (95% CI: 96.4–97.8) with no significant difference between males and females (Table 6).

**Table 6:** Prevalence of physical inactivity stratified by gender.

Risk factors	Total	Males (*n* = 777)	Females (*n* = 1,623)	*P*-value for trend (males vs. females)
	% (95% CI)	% (95% CI)	% (95% CI)	
Physical activity				
Low (<600 MET-min)	97.2 (96.4–97.8)	97.2 (95.7–98.1)	97.2 (96.2–97.9)	0.997

Table 7 illustrates self-reported raised blood sugar levels, which may indicate prediabetes or diabetes. The prevalence is generally higher in middle-aged individuals (45–54 years) and then declines in older age groups (≥66 years). However, there was no significant difference across the age groups.

**Table 7:** The prevalence of self-reported raised blood sugar levels by age group.

	Raised blood sugar levels						
Age Gender	18–24 years	25–34 years	35–44 years	45–54 years	55–64 years	≥66 years	*p*-value
	*n* (%)	*n* (%)	*n* (%)	*n* (%)	*n* (%)	*n* (%)	
Females	38 (4.6)	135 (16.3)	189 (22.9)	222 (26.8)	172 (20.8)	71 (8.6)	0.262
Males	30 (8.0)	49 (13.1)	96 (25.6)	96 (25.6)	79 (21.1)	25 (6.7)	0.850

Table 8 presents self-reported raised blood pressure levels, which are an indicator of hypertension. The prevalence increases with age, peaking at 45–54 years, before declining in the oldest age group with no significant difference across age groups.

**Table 8:** The prevalence of self-reported raised blood pressure levels by age group.

	Raised blood pressure levels						
Age Gender	18–24 years	25–34 years	35–44 years	45–54 years	55–64 years	≥66 years	*p*-value
	*n* (%)	*n* (%)	*n* (%)	*n* (%)	*n* (%)	*n* (%)	
Females	43 (5.2)	122 (14.8)	197 (23.9)	204 (24.7)	190 (23.0)	70 (8.5)	0.350
Males	30 (7.5)	53 (13.3)	103 (25.9)	106 (26.6)	81 (20.4)	25 (6.3)	0.880

Table 9 below presents the determinants of behavioral risk factors for NCDs and it reveals that age was statistically significantly associated with behavioral risk factors for NCDs as older participants (≥35 years) were likely to have low fruit and vegetable intake (aOR = 1.3; 95% CI: 0.99–1.62). Marital status was also statistically significantly associated with behavioral risk factors for Widows were 30% less likely to smoke (aOR = 0.72; 95% CI: 0.57–0.92), yet they were 1.4 times more likely to consume alcohol (aOR = 1.4; 95% CI: 0.99–1.84). Participants who were unemployed or not working were found to have a higher likelihood of consuming alcohol (aOR = 1.3; 95% CI: 1.02–1.54). Gender and educational status were not statistically significantly associated with behavioral risk factors for NCDs.

**Table 9:** Multivariate logistic regression to determine predictors of behavioral risk factors for NCD.

Variables	Smoking	Alcohol consumption	Low fruit and vegetable intake	Physical inactivity	Raised BP	Raised sugar level
Age						
18–34 years	Reference (1)	Reference (1)	Reference (1)	Reference (1)	Reference (1)	Reference (1)
≥35 years	1.1 (0.89–1.32)	1.2 (0.96–1.56)	1.3 (0.99–1.62)*	0.75 (0.39–1.44)	1.1 (0.83–1.23)	0.9 (0.76–1.13)
Gender						
Female	Reference (1)	Reference (1)	Reference (1)	Reference (1)	Reference (1)	Reference (1)
Male	1.2 (0.84–1.34)	0.97 (0.78–1.21)	0.97 (0.79–1.18)	0.99 (0.59–1.67)	0.9 (0.76–1.06)	1.1 (0. 85–1.20)
Educational status						
High	Reference (1)	Reference (1)	Reference (1)	Reference (1)	Reference (1)	Reference (1)
Low	1.1 (0.89–1.22)	0.96 (0.78–1.18)	1.1 (0.87–1.27)	0.9 (0.61–1.61)	0.9 (0.75–1.05)	0.9 (0.85–1.17)
Marital status						
Married	Reference (1)	Reference (1)	Reference (1)	Reference (1)	Reference (1)	Reference (1)
Never married	0.85 (0.69–1.05)	1.2 (0.92–1.60)	0.9 (0.74–1.22)	0.6 (0.29–1.16)	0.84 (0.61–1.05)	0.9 (0.78–1.19)
Divorced	1.1 (0.79–1.26)	1.2 (0.86–1.56)	1.1 (0.79–1.36)	0.9 (0.49–1.78)	0.9 (0.74–1.17)	1.1 (0.78–1.19)
Widowed	0.72 (0.57–0.92)*	1.4 (0.99–1.84)*	1.1 (0.75–1.33)	0.9 (0.49–1.91)	0.86 (0.57–0.92)	0.9 (0.78–1.27)
Work status						
Working	Reference (1)	Reference (1)	Reference (1)	Reference (1)	Reference (1)	Reference (1)
Not working	1.1 (0.90–1.25)	1.3 (1.02–1.54)*	0.9 (0.80–1.17)	0.6 (0.39–1.03)	0.9 (0.81–1.11)	0.9 (0.81–1.11)

Values are reported as odds ratios (95% CI).

* Significant *at p < 0.05.*

** Significant *at p < 0.005.*

*** Significant *at p < 0.001* a = Not significant in univariate model then dropped.

## 4 Discussion

The study aimed to determine the prevalence and determinants of behavioral risk factors contributing to NCDs in Bushbuckridge, Ehlanzeni District Municipality of Mpumalanga Province. The study consisted of 2,400 participants with a mean age of 46.27 ± 13.38, with the majority of the participants being females as compared to males. Similar findings have been reported in other studies, where female participation often exceeds that of males (20–22). This trend is commonly attributed to women being more likely to seek and utilize healthcare services, possess greater health knowledge, adhere to medical programs, and prioritize both their health and that of others (20, 23). Additionally, men are more likely to be employed as day labourers or hold formal jobs, making it difficult for them to participate in studies conducted during the day (24).

In the total population of the present study, the prevalence of smoking was 51.3% with no significant difference in smoking prevalence between males and females. In contrast with the findings of the present study, previous studies reported smoking to be more common in males as compared to females (21, 25). The inconsistency between the findings of the present study and previous studies may be due to the difference in the study setting and different geographic locations. Furthermore, the findings of the present study noted that the majority of participants began smoking between 18 and 25 years old, accounting for 30.7%. Those who started between 36 and 45 years old at 17.3% and those who began followed this between 46 and 55 years old at 3%. Several studies have found trends in smoking initiation similar to the findings of the present study, particularly among young adults (26, 27). Furthermore, another study observed a shift where cigarette initiation, which traditionally peaked during teenage years, is now extending into early adulthood (ages 18–23), partly due to the influence of lifestyle changes and reduced anti-smoking interventions targeted at this older demographic (28). This shift could be linked to socioeconomic pressures, lifestyle changes, or changing cultural norms around smoking. These findings indicate a need for targeted smoking prevention programs that extend beyond adolescence to curb the growing trend of later initiation ages.

The prevalence of those who consumed alcoholic drinks in the last 12 months was 19.3 with no statistically significant difference between males and females. In addition, on multivariate regression, there was no association between gender and alcohol consumption. In contrast with the findings of the present study, previous studies noted males have a higher prevalence of alcohol consumption when compared to their female counterparts (21, 25). The inconsistencies between the findings of the present study and previous studies may be due to that traditional gender roles in many rural areas, including Bushbuckridge, might be evolving (29). While historically, alcohol consumption was often seen as more acceptable for males (29), changing social dynamics could lead to greater parity in drinking habits. This may be particularly true in more mixed-gender environments where people have more freedom and social acceptance to engage in similar behaviours. Moreover, in areas with economic challenges, both men and women may drink alcohol as a coping mechanism for stress or as part of socializing. Poverty, unemployment, and other economic pressures can affect both genders equally, leading to similar drinking patterns (30–32). Furthermore, the above findings are in agreement with the findings of the present study, on multivariate regression we noted unemployed and participants with deceased partners were more likely to consume alcohol.

In the present study, the prevalence of inadequate fruit and vegetable consumption was 76.2%. The findings of the present study are in alignment with the studies conducted in South Asia and Sub-Saharan Africa, which reported that over 70% of the population often falls short of dietary guidelines (33–36). These findings call for a need for public health strategies to increase fruit and vegetable availability and affordability, as well as public awareness campaigns promoting healthy dietary habits. In addition, on multivariate logistic regression participants aged ≥35 years were 1.3 times more likely to have low fruit and vegetables intake. In agreement with the findings of the present study, Mehranfar et al. (37), indicated that older adults are particularly vulnerable to low fruit and vegetable consumption, which is linked to a higher risk of chronic diseases, such as hypertension and diabetes.

Furthermore, the findings of the study revealed a high prevalence of physical inactivity, affecting 97.2% of the population, with no notable variation between males and females. The findings of the present study reported a higher proportion of physical inactivity compared to a study by Aftab et al. found low physical inactivity. These findings highlight the urgent need to address the high prevalence of physical inactivity, as Mtintsilana et al. (38), point out that insufficient physical activity has been a significant factor contributing to NCDs like hypertension and diabetes.

In the present study, the prevalence of hypertension was reported to be 51%, with no significant difference between males and females. The present study noted a higher prevalence of hypertension as compared to other studies reported in rural South Africa that ranged between 25% to 36% (21, 39, 40). The difference between the findings of the present study and the previous studies may be the difference in study design. In the present study, hypertension was identified based on self-reported data, whereas previous studies diagnosed hypertension by reviewing individual medical histories and measuring blood pressure. Furthermore, individuals with a systolic blood pressure of ≥140 mmHg, a diastolic blood pressure of ≥90 mmHg, or both were classified as hypertensive in those earlier studies.

In addition, when stratified by age categories participants aged 35–44, 45–54, and 55–64 in both males and females were noted to have the highest prevalence of hypertension as compared to other age groups. This may have been attributed to the overrepresentation of participants in those particular age groups. Additionally on regression, although not statistically significant, participants aged 35 and above were 1.1 times more likely to be associated with hypertension. However, previous studies reported the increase in age to be associated with hypertension (25, 41, 42).

Previous studies conducted in South Africa reported the prevalence of diabetes to range between 8%–11%, affecting females as compared to males (43–46). In addition, studies conducted in rural South Africa noted the same prevalence (21, 40). In contrast with the previous studies, the findings of the present study noted a higher prevalence of diabetes of 50.1%, with no significant differences between males and females. The variation between the findings of this study and previous research could be attributed to differences in study design and different geographic locations. Additionally, in the current study, diabetes was identified using self-reported data.

### Study limitations

Due to the design being cross-sectional, the study could not assess causal relationships. The findings may not be a full representative of the general community as the participants were recruited in the healthcare facility. The study utilized self-reported data to assess key behavioral risk factors such as smoking, alcohol consumption, dietary habits, physical inactivity and raised blood pressure and raised blood sugar. While self-reported data may be subject to recall bias, it remains an important method for capturing behavioral patterns in the population. Despite these limitations, we believe this research offers valuable insights into the prevalence and determinants of behavioral risk factors contributing to NCDs in Bushbuckridge.

## 5 Conclusion

The prevalence of behavioral risk factors for NCDs was found to be high among rural populations residing in Bushbuckridge. Underscoring the need for sustained and comprehensive interventions. In rural areas like Bushbuckridge, the combination of poverty, unemployment, limited healthcare access, and evolving social dynamics creates a challenging environment that fosters unhealthy behaviors and increases the risk of NCDs. To effectively reduce the burden of these diseases in such communities, public health strategies must focus on socio-economic and cultural determinants, rather than just demographic factors. These strategies could include community-based campaigns, workshops with local leaders, and initiatives targeting lifestyle factors such as poor diets, physical inactivity, alcohol use, and smoking, all aimed at fostering healthier communities.

## Data Availability

The raw data supporting the conclusions of this article will be made available by the authors, without undue reservation.
