# Supplementary material for: The prevalence and behavioral risk factors contributing to non-communicable diseases in Bushbuckridge, Mpumalanga province, South Africa
Source: Front Epidemiol. 2025 Apr 10;5:1560971. doi: 10.3389/fepid.2025.1560971 (PMC12018342; doi:10.3389/fepid.2025.1560971)
Supplement: Supplementary file 1 [file Table1.docx]

**Consent form**

I have understood the aims and objectives of the proposed study and I was granted opportunity to ask any questions prior to the study. The aim and objectives of the study are sufficiently clear to me. I have not been forced to participate in study.

I understand that participation in this study is voluntary and that I may withdraw from it at any time without any harm.

I know that this study has been approved by the Turfloop Research Ethics Committee (TREC), University of Limpopo (Turfloop Campus). I am fully aware that the results of the study will be used for scientific purposes and may be published. I agree to this, provided my privacy and confidentiality will be maintained.

I hereby give consent to participate in this study.

Signature of Participant____________________________________________

Date................................Place.................................... Witness…………………………….

_____________________________________________________________________

Statement by the Researcher

I will maintain privacy and confidentiality as promised.

.

Name of Researcher: Pilusa Thabo Signature…………………Date…………………….


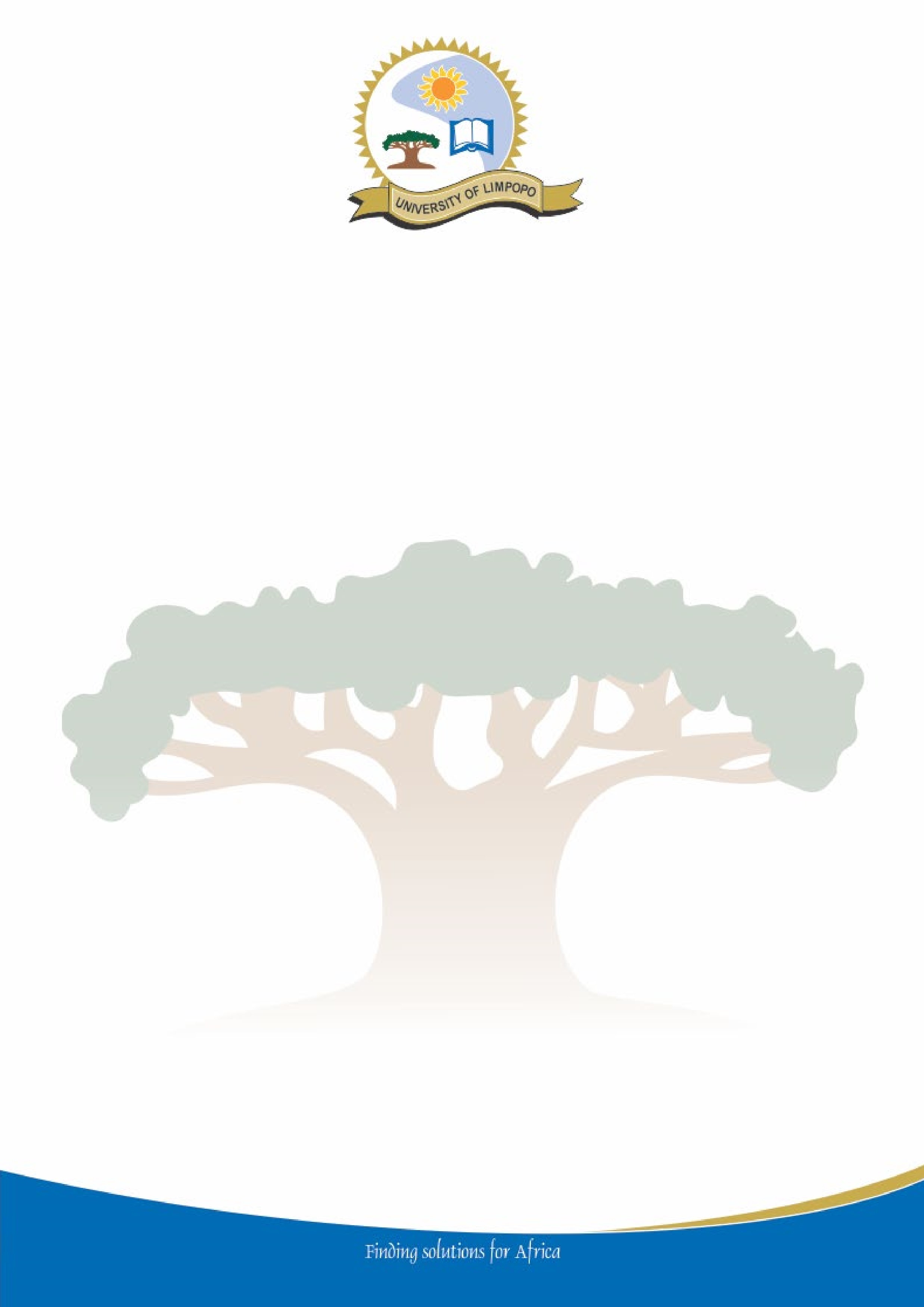


**University of Limpopo**

**Department of Research Administration and Development**

**Private Bag X1106, Sovenga, 0727, South Africa**

**Tel: (015) 268 3935, Fax: (015) 268 2306, Email: tukiso.sewapa@ul.ac.za**

**MEETING:**

**04**

**December 2023**

**PROJECT NUMBER:**

**TREC/1777/2023: PG**

**PROJECT:**

**Title:**

Developing Strategies to Prevent Behavioural Risk Factors for Hypertension and

Diabetes in Bushbuckridge, Ehlanzeni District, Mpumalanga Province, South

Africa

**Researcher:**

TD Pilusa

**Supervisor:**

Prof E Maimela

**Co-Supervisor/s:**

N/A

**School:**

Health Care Sciences

**Degree:**

PhD Public Health

**PROF D MAPOSA**

**CHAIRPERSON: TURFLOOP RESEARCH ETHICS COMMITTEE**

The Turfloop Research Ethics Committee (TREC) is registered with the National Health Research Ethics

Council, Registration Number:

**REC-0310111-031**


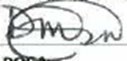


**Note:**

**i)**

**This Ethics Clearance Certificate will be valid for one (1) year, as from the abovementioned date.**

**Application for annual renewal (or annual review) need to be received by TREC one month**

**before lapse of this period.**

**ii)**

**Should any departure be contemplated from the research procedure as approved, the**

**researcher(s) must re-submit the protocol to the committee, together with the Application for**

**Amendment form.**

**iii)**

**PLEASE QUOTE THE PROTOCOL NUMBER IN ALL ENQUIRIES.**

**TURFLOOP RESEARCH ETHICS**

**COMMITTEE**

**ETHICS CLEARANCE CERTIFICATE**

**APPENDIX I**


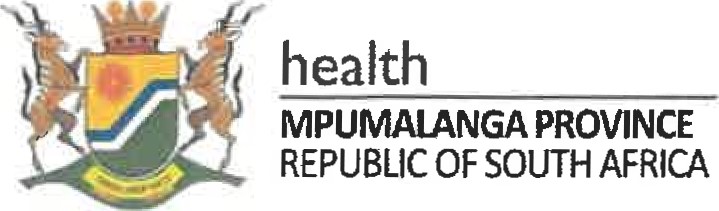
MPUMALANGA

THE PLACE OF THE atSiHG SUN

No.3, Government Boulevard, Riverside Park, Ext. 2, Mbombela, 1200, Mpumalanga Province

Private Bag Xl 1285, Mbombela, 1200, Mpumalanga Province Tel l: +27 (13) 766 3429, Fax: +27 (13) 766 3458

Litiko Letemphilo Departement van Gesondheid UmNyango WezeMaphilo


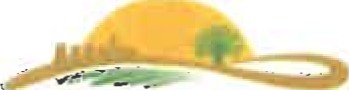
Letter of Support Signed by Chief Director (CD)/CEO/District Manager (DM)/Programme Manager (PM)

| 1. Name & contact no. of Applicant | PILUSA THABO DIFFERENCE | | | |
| --- | --- | --- | --- | --- |
| 2. Title of Study: DEVELOPING STRATEGIES TO PREVENT BEHAVIORAL RISK FACTORS FOR HYPERTENSION  AND DIABETES IN BUSHBUCKRIDGE, EHLANZENI DISTRICT, MPUMALANGA PROVINCE, SOUTH AFRICA | | | | |
| 3. Aim and population target: The aim of the study is to develop strategies to improve prevention, control of behavioural risk factors contributing to non-communicable diseases in Bushbuckridge, Ehlanzeni District, Mpumalanga Province, South frica. Target group: health care workers, community workers and patients. | | | | |
| 4. Period to undertake the study | From: December 2023 to: December 2024 | | | |
| 5. Resources equire from Facility | | | | |
| 5.1: Facility Staff Required to assis with the Study | Yes x | | | NO |
|  | How many: | | |  |
|  | Nurses: | 24 | |  |
|  | Doctors: |  | |  |
|  | Other, please specify: | 8 | |  |
| 5.2: Patient Records/Files | Yes | | | NO x |
| 5.3: Interviewing Patient at Facilities | Yes x | | | NO |
| 5.4: Interviewing Patients at Home | Yes | | | NO x |
| 5.5:Resource Flow (Are there benefits to Patients/community) | Yes x | | | NO |
|  | Please list: HPT & DM risk behavioural factors awareness. | | |  |
| 5.6: Resource Flow (Are there benefits to Facility/District)   \| 6 Availability of Required Clearance \| \| --- \| | Yes x | | | NO |
|  | Please list: Strengthening prevention and control of behavioural risk factors for HTP & DM. | | |  |
|  | 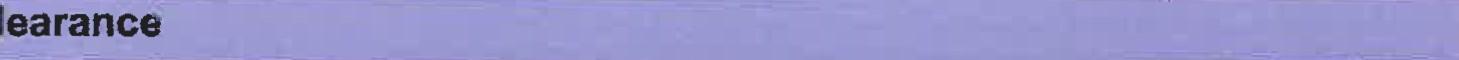 | | | |
| 6.1: Ethical Clearance | Yes X | | Pending | NO |
|  | Clearance Number: TREC/177712023:PG | |  |  |
| 6.2: Clinical Trial | Yes | | Pending | NOX |
|  | Clearance Number: | |  |  |
| 6.3: Vaccine Trial | Yes | | Pending | NOX |
|  | Clearance Number: | |  |  |
| 6.4: Budget  Declaration by Applicant:  I Mr/Ms/Dr/Prof/Adv. Pilusa Thabo CEO/lnstitution/District. | Yes X | | | NO  back to the |
|  | Source of fund: Self-funding  Difference agree to submit/present the result of this study | | |  |


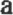
Please note that this letter is not an approval to undertake i.e.
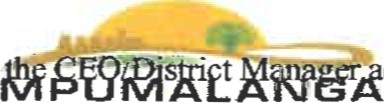
cknowledges to have been a study, but support letter from consulted on the studyidentified facilit THE AACE OF THE y/ðNnct.SUN

| 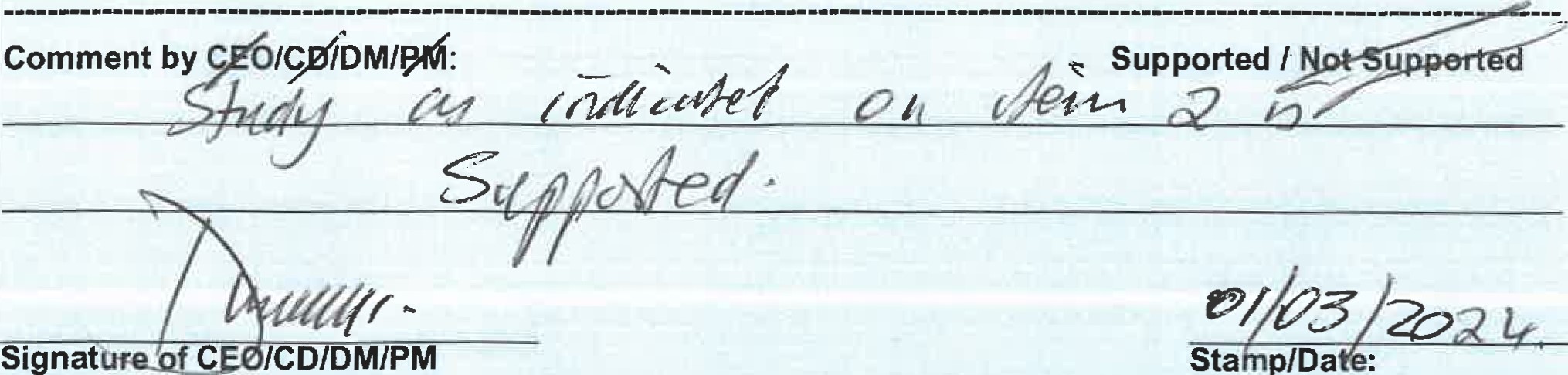 Name: | | | | | | |
| --- | --- | --- | --- | --- | --- | --- |
|  |  |  |  |  |  |  |
| Please email completed form to: JerryS@mpuhealth.qov.za ThembaM@mpuhealth.gov.za | | | | | | |
| 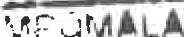 | |  | 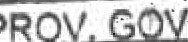 | RNM NT |  |  |
| OtSTRtCT  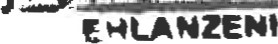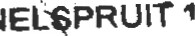PRIVATE BAG X 11278  ^A^ NDERSON STREET 20  D.ììe 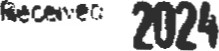 -03- 01  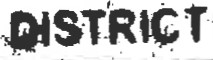MANAGERS OFFICE | | | | |  |  |
|  | EPARTMENT OF HEALTH | | | |  |  |

**APPENDIX J**


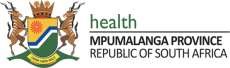


Indwe Building, Government Boulevard, Riverside Park, Ext. 2, Mbombela, 1200, Mpumalanga Province Private Bag X11285, Mbombela, 1200, Mpumalanga Province

Tel l: +27 (13) 766 3429, Fax: +27 (13) 766 3458

Litiko Letemphilo Departement van Gesondheid UmNyango WezeMaphilo

Enq: 013 766 3766

Ref: MP_202403_001

**Research Permission Letter**

**MR T PILUSA**

**PRINCIPAL INVESTIGATOR**

**PO BOX 3467**

**ACORNHOEK 1360**

**Dear Mr Pilusa**

***STUDY TITLE: DEVELOPING STRATEGIES TO PREVENT BEHAVIORAL RISK FACTORS FOR HYPERTENSION AND DIABETES IN BUSHBUCKRIDGE, EHLANZENI DISTRICT, MPUMALANGA PROVINCE, SOUTH AFRICA***

The Mpumalanga Provincial Health Research and Ethics Committee (MPHREC) has accepted your research proposal in the latest format you sent, and hereby grant you permission to conduct your research as detailed below.

-
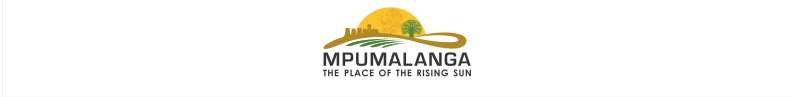
Approval Reference Number: **MP_202403_001**
- Data Collection Period: **10/03/2024 to 30/12/2024**
- Approved Data Collection Facilities: **ARTHURSEAT CLINIC; BROOKLYN CLINIC;**

**BUFFELSHOEK CLINIC; COTTONDALE CLINIC;**

**MOREIPUSO CLINIC; MURHOTSO CLINIC**

Kindly ensure that conditions mentioned below are adhered to, and that the study is conducted with minimal disruption and impact on our staff, and also ensure that you provide us with a soft or hard copy of the report once your research project has been completed.

***Conditions:***

- *Researchers not allowed to make copies, take pictures of medical records or administer medicine to patients at the facility.*
- *Kindly notify the facility manager* ***a week BEFORE*** *you start with data collection to ensure that conditions are conducive in the facility.*
- *The FINAL RESEARCH FINDINGS must be uploaded on the NHRD website.*


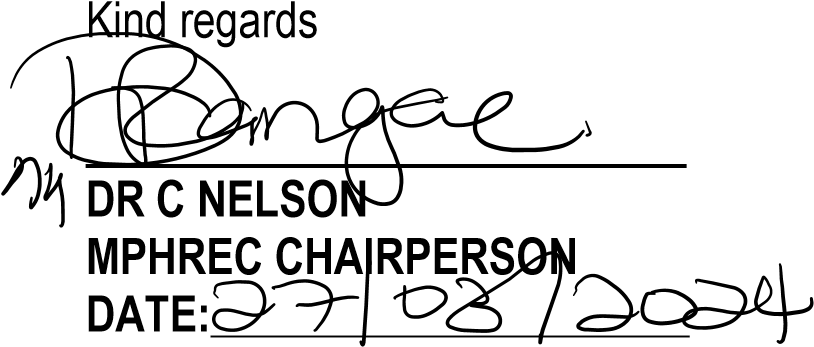


**WHO STEPS Instrument**

<SOUTH AFRICA, MPUMALANGA PROVINCE / BUSHBUCKRIGDE SUB-DISTRICT/COTTONDALE LOCAL AREA

| **Survey Information / TshedimoŠo ka ga dinyakiŠiŠo** |
| --- |

| **Please** | **A** | **B** | **C** | **D** | **E** | **F** |
| --- | --- | --- | --- | --- | --- | --- |
|  |  |  |  |  |  |  |

FACILITY:

**SECTION A: DEMOGRAPHIC INFORMATION/ TSHEDIMOŠO YA TŠA BODULO**

| **NO** | **Question/*Po*tšiso** | **Answer/Karabo** | **Code** |
| --- | --- | --- | --- |
| 1 | Sex (*Record Male / Female as observed) /*  Bong (*Monna/ Mosadi)* | Male/Monna 1  Female/Mosadi 2 | C1 |
| 2 | Age / Letšatši la Mengwaga? |  | C2 |
| 3 | Educational status */*  Tša dithuto | Matric/Marematlou 1  Primary/mphato wa fase 2  Tertiary /Mphato wa gedimo 3  Never schooled/ a ka tsena sekolo 4 | C3 |
| 4 | Employement/  Mošomo | Employed/ka šoma 1  Unemployed/ a ke šome 2 | C4 |
| 5 | What is your **marital status**? / Maemo a gago a lenyalo ke eng? | Never married / Ga se ka nyala/nyalwa 1  Currently married / Ke nyetše/nyetšwe 2  Divorced / Re kgaogane ka semolao 4  Widowed / Ke hlokofaletswe ke molekane 5 | C5 |
| 6 | What is your  *ethnic group* ?  Naa o mohlobo mang (go ya ka **polelo**/ **setso**) | Pedi 1  Tsonga 2  Other 3 | C6 |

**SECTION B: BEHAVIOURAL RISK FACTORS**

**TOBACCO USE /TŠHUMIŠO YA MOTSOKO**

| **CORE: TOBACCO USE / DIPOTŠISO TŠE BOHLOKWA: Tšhumišo ya Motsoko** |
| --- |
| Now I am going to ask you some questions about various health behaviours. This includes things like smoking, drinking alcohol, eating fruits and vegetables and physical activity. Let's start with tobacco. /  Bjale ke tlo go botšiša dipotšišo mabapi le tsa maphelo. Go akaretša go kgoga, go nwa bjala, go ja dienywa le merogo le go thobolla mmele. A re thome ka ka motsoko. |

| **NO** | **Question/*Po*tšiso** | **Answer/Karabo** | **Code** |
| --- | --- | --- | --- |
| 7 | Do you currently smoke any **tobacco products**, such as cigarettes, cigars or pipes?  Naa gona bjale o kgoga e mengwe ya metšoko ya go swana le sekerete, sikara goba peipe? | Yes/Ee 1  No/Aowa 2 | T1 |
| 8 | Do you currently smoke tobacco products **daily**?  Naa le kgoga mehuta ye tšatši le tšatši? | **If answer is NO to above question thick N/A from T2-T6.**  Yes/Ee 1  No/Aowa 2  N/A 3 | T2 |
| 9 | Do you consider stop to the use of tobacco?  Na o ikemiseditse go kgaotša go fola motsoko ? | Yes/Ee 1  No/Aowa 2  N/A 3 | T3 |
| 10 | Do you easily access tobacco or snuff?  Naa o kgwetša motsoko ga bonolo? | Yes/Ee 1  No/Aowa 2  N/A 3 | T4 |
| 11 | What influence you to start smoking?  O tutueditse ke eng go kgoga motsoko? | Peer pressure/Bagwera 1  Family member/ wa leloko 2  Boredom/ go jewa ke bodutu 3  N/A 4 | T5 |
| 12 | How old were you when you **first started** smoking tobacco?  Naa o be o na le mengwaga e me kae **ge o thoma** go kgoga? | Age 18-35/ magareng ga 18-35 ya mengwaga 1  Age 36-45/ magareng ga 18-35 ya mengwaga 2  Age 46-55/ magareng ga 18-35 ya mengwaga 3  Age 56-65/ magareng ga 18-35 ya mengwaga 4  N/A 5 | T6 |
| 13 | Is there someone at home who is smoking? | Yes/Ee 1  No/Aowa 2 | T7 |
| 14 | During the past 7 days, on how many days did someone **in your home** smoke when you were present?  Mo matšatšing a 7 a go feta, naa go na le matšatši a makae mo go ilego gwa ba le yo mongwe **ka lapeng** yo a ilego a fola o le gona? | 7 days/ matšaši a šupa 1  5-6 days per week/Matšaši a 5-6 ka beke 2  1-4 days per week/ Matšaši i a 1-4 ka beke 3  N/A 4 | T8 |
| 15 | Do you know the danger of smoking? Na o tseba kotsi ya go kgoga? | Yes/Ee 1  No/Aowa 2 | T9 |

**ALCOHOL CONSUMPTION / TŠHOMIŠO YA BJALA**

| **CORE: ALCOHOL CONSUMPTION / DIPOTŠIŠO TŠE BOHLOKWA: Tšhomišo ya Bjala** |
| --- |
| The next questions ask about the consumption of alcohol./ Dipotšišo tša go latela di a mana le tšhomišo ya bjala. |

| **NO** | **Question/*Po*tšišo** | **Answer/Karabo** | **Code** |
| --- | --- | --- | --- |
| 16 | Have you **ever** consumed an alcoholic drink such as beer, wine, spirits, fermented cider or *traditionally fermented beer*?  Naa o ile wa nwa bjala bjalo ka biri, beine, dino tša bogale re bala le thothotho, disaita (“ciders”) goba bjala bja Sesotho? | Yes/Ee 1  No/Aowa 2 | A1 |
| 17 | **If yes to the above question**  Have you consumed an alcoholic drink within the **past 12 months**? /  Naa o ile wa nwa bjala mo kgweding tše lesome pedi (12) tša go feta? | **If answer is NO to above question thick N/A from A2-A8.**  Yes/Ee 1  No/Aowa 2  N/A 3 | A2 |
| 18 | During the past 12 months, **how frequently** have you had at least one alcoholic drink? /  Mo dikgweding tše lesome pedi (12) tša go feta, naa o ile wa nwa bjala makga a ma kae? | Daily/ Tšaši ka tšaši 1  5-6 days per week/Matšaši a 5-6 ka beke 2  1-4 days per week/ Matšaši i a 1-4 ka beke 3  Less than once a month// Ka tlase ga ga-tee ka kgwedi 4  N/A 5 | A3 |
| 19 | During the past 30 days, when you consumed an alcoholic drink, how often was it with meals? Please do not count snacks.  Mo matšatšing a masome tharo (30) a go feta, moo obego o enwa bjala, ke ga kae moo obego o bo nwa ebile o eja? Hlokomela gore o se bale diseneks (snacks). | Usually with meals /Le dijo ka mehla 1  Sometimes with meals / Le dijo ka nako tše dingwe 2  Rarely with meals / Ga se gantši 3  Never with meals / Ntle le dijo 4  N/A 5 | A4 |
| 20 | Do you consider stopping alcohol consumption?  Na o ikemiseditse go kgaotša  go nwa bjala? | Yes/Ee 1  No/Aowa 2  N/A 3 | A5 |
| 21 | What influence you to start consuming alcohol?  O tutueditse ke eng go nwa bjala? | Peer pressure/Bagwera 1  Family member/ wa leloko 2  Boredom/ go jewa ke bodutu 3  N/A 4 | A6 |
| 22 | Do you have an easy access to alcohol outlets?  Na o hwetša bjala ga bonolo? | Yes/Ee 1  No/Aowa 2  N/A 3 | A7 |
| 23 | How old were you when you **first started** consuming alcohol?  Naa o be o na le mengwaga e me kae **ge o thoma** go nwa bjala? | Age 18-35/ magareng ga 18-35 ya mengwaga 1  Age 36-45/ magareng ga 18-35 ya mengwaga 2  Age 46-55/ magareng ga 18-35 ya mengwaga 3  Age 56-65/ magareng ga 18-35 ya mengwaga 4  N/A 5 | A8 |
| 24 | Do you know the danger of alcohol consuptions? Na o tseba kotsi ya go nwa bjala? | Yes/Ee 1  No/Aowa 2 | A9 |

**DIET / TŠA DIJO**

| **CORE: DIET / DIPOTŠIŠO TŠA BOHLOKWA: Tša Dijo** |
| --- |
|  |

| **NO** | **Question/*Po*tšiso** | **Answer/Karabo** | **Code** |
| --- | --- | --- | --- |
| 25 | In a typical week, on how many days do you **eat fruit**?  Mo bekeng yeo e tlwaelegilego, na o **ja dienywa** matšatši a makae? | Daily/ Tšaši ka tšaši 1  5-6 days per week/Matšaši a 5-6 ka beke 2  1-4 days per week/ Matšaši i a 1-4 ka beke 3  Not at all/a ke di ji? 4 | D1 |
| 26 | In a typical week, on how many days do you **eat vegetables**?  Mo bekeng yeo e tlwaelegilego, na o **ja merogo** matšatši a makae? | Daily/ Tšaši ka tšaši 1  5-6 days per week/Matšaši a 5-6 ka beke 2  1-4 days per week/ Matšaši i a 1-4 ka beke 3  Not at all/a ke di ji? 4 | D2 |
| 27 | Do you take excessive fat diet/?  Na o ja tša makhura a mantši dijong? | Yes/Ee 1  No/Aowa 2 | D 3 |
| 28 | What type of **oil or fat is most often** used for meal preparation in your household?  Naa o šomiša **makhura** a mohuta mang ge o apea? | Vegetable oil / Makhura a merogo 1  Butter or ghee / Botoro 2  Margarine / Margarine 3  Other / A mangwe 4  None used / Ga ke šomiše Makhura 5 | D4 |
| 29 | On average, how many meals per week do you eat that were not prepared at a home? By meal, I mean breakfast, lunch and dinner.  Go ya ka palogare, naa o ja dijo tše kae tšeo di sa apewago ka gae? Ke ra gore difihlolo, matena le dilalelo. | Daily/ Tšaši ka tšaši 1  5-6 days per week/Matšaši a 5-6 ka beke 2  1-4 days per week/ Matšaši i a 1-4 ka beke 3  Less than once a month// Ka tlase ga ga-tee ka kgwedi 4 | D5 |
| 30 | Do you take excessive salt diet/?  Na o ja tša letswai le ntši dijong? | Yes/Ee 1  No/Aowa 2 | D6 |
| 31 | Do you take excessive sugar/?  Na o ja swikiri entši dijong? | Yes/Ee 1  No/Aowa 2 | D7 |
| 32 | Do you take fizzy drinks?  Na o nwa di nwa maphodi? | Yes/Ee 1  No/Aowa 2 | D8 |

**PHYSICAL ACTIVITY / THOBOLLO YA MMELE**

| **CORE: PHYSICAL ACTIVITY / DIPOTŠITŠO TŠE BOHLOKWA: Thobollo ya mmele** |
| --- |
| Next I am going to ask you about the time you spend doing different types of physical activity in a typical week. Please answer these questions even if you do not consider yourself to be a physically active person.  Think first about the time you spend doing work. Think of work as the things that you have to do such as paid or unpaid work, study/training, household chores, harvesting food/crops, fishing or hunting for food, seeking employment. *[Insert other examples if needed].* In answering the following questions 'vigorous-intensity activities' are activities that require hard physical effort and cause large increases in breathing or heart rate, 'moderate-intensity activities' are activities that require moderate physical effort and cause small increases in breathing or heart rate. /  Bjale ke ile go go botšiša ka nako yeo o e tseyago o thobolla mmele mo bekeng. Ka kgopelo araba dipotšišo le ge o ša ipone o le motho wa go fela a ithobolla mmele. Nagana pele ka nako yeo o etšeago o soma. Nagana ka mošomo ele dilo tšeo o di dirago go swana le mošomo wa go lefšwa goba wa go se lefšwe, go ithuta, mešomo ya ka gae, go lema, go thea dihlapi goba go nyakana le mošomo.[Tsentšha mehlala ye mengwe ge go hlokega]. Ge o fetola dipotšišo tše latelago;tseba gore ge re bolela ka mošomo o boima re ra gore mošomo woo o dirago gore o hemele godimo le pelo e kibela godimo, mola mošomo o boleta e le woo o dirago gore o se hemele godimo kudu le pelo e se kibela godimo kudu. |

| **NO** | **Question/*Po*tšišo** | **Answer/Karabo** | **Code** |
| --- | --- | --- | --- |
| 33 | Do you do any vigorous-intensity sports, fitness or recreational *(leisure)* activities that cause large increases in breathing or heart rate like *[running or football]*  for at least 10 minutes continuously?  A naa o tšea karolo mo dipapading tšeboima, tša boitekanelo goba tša boitapološo (boiketlo) tšeo di fegedišago goba di dirago gore pelo e kibele godimo (go kitima goba go raloka kgwele ya maoto ) tekano ya metsotso e lesome. | Yes/Ee 1  No/Aowa 2 | P1 |
| 34 | Do you walk or use a bicycle *(pedal cycle)* for at least 10 minutes continuously to get to and from places? / Naa o a sepela goba o šomisa paesekela (ya materapo) go lekana nako ya metsotso e lesome goba go feta ge o eya goba o bowa mafelong? | Yes/Ee 1  No/Aowa 2 | P2 |
| 35 | How much time do you spend doing vigorous-intensity activities at work on a typical day? /  Naa o tšea nako e kaakang ge o šoma boima mo letšatšng le le tee? | 5 minutes/5 ya metsotso 1  10 Minutes/10 ya metsotso 2  30 Minutes/30 ya metsotso 3  60 minutes/ 60 ya metsotso 4 | P3 |
| 36 | In a typical week, on how many days do you do vigorous-intensity activities as part of your work? /  Mo bekeng ye e tlwaelegilego, ke matšatši a ma kae o šoma boima. | Daily/ Tšaši ka tšaši 1  5-6 days per week/Matšaši a 5-6 ka beke 2  1-4 days per week/ Matšaši i a 1-4 ka beke 3  1-3 days per week// Matšaši a 1-3 ka beke 4 | P4 |

**SECTION C: HEALTH FACILITY FACTORS**

| **HISTORY OF RAISED BLOOD PRESSURE / DIPOTŠITŠO TŠE BOHLOKWA: TŠA MADI A MAGOLO** |
| --- |

| **NO** | **Question/*Po*tšišo** | **Answer/Karabo** | **Code** |
| --- | --- | --- | --- |
| 37 | Have you ever had your blood pressure measured by a doctor or other health worker?  A naa o ile wa bofša lepanta la tekolo ya boima bja kelo ya madi ke ngaka goba mošomedi o mongwe wa tša maphelo? | Yes/Ee 1  No/Aowa 2 | H1 |
| 38 | Have you ever been told by a doctor or other health worker that you have raised blood pressure or hypertension?  A naa o kile wa botšwa ke ngaka goba mošomedi o mongwe wa tsa maphelo gore boima bja kelo ya madi bo goletše godimo goba o na le madi a magolo? | Yes/Ee 1  No/Aowa 2 | H2 |
| 39 | Are there drugs (medication) that you have taken in the past two weeks?  Na o tsere diokobatši (dihlare ) tšeo o di nwelego mo dibekeng tše pedi tša gofeta? | Yes/Ee 1  No/Aowa 2 | H3 |
| 40 | Have you adviced to reduce salt intake?  Na o kile wa eletšwa go fokotša letswai dijong? | Yes/Ee 1  No/Aowa 2 | H4 |
| 41 | Have you received an advice or treatment to lose weight?  Na o kile wa newa Keletšo goba kalafo ya go fokotša boima bja mmele? | Yes/Ee 1  No/Aowa 2 | H5 |
| 42 | Have you received an advice or treatment to stop smoking?  Na o kile wa newa Keletšo goba kalafo ya go tlogela go kgoga motšoko? | Yes/Ee 1  No/Aowa 2 | H6 |
| 43 | Have you received an advice to start or do more exercise?  Na o kile wa newa Keletšo ya go thoma go itšhidulla kudu | Yes/Ee 1  No/Aowa 2  N/A 3 | H7 |
| 44 | Have you ever seen a traditional healer for raised blood pressure or hypertension? /  A naa o ile wa bonwa ke ngaka ya setšo mabapi le madi a magolo? | Yes/Ee 1  No/Aowa 2 | H8 |
| 45 | Any knowledge of behavioural risk factors for hypertension?  A naa o tseba mekgwa yeo e bakago bolwetši madi ao a magolo ? | Yes/Ee 1  No/Aowa 2  N/A 3 | H9 |
| 46 | Have you ever experience shortage of Hypertension treatment in the facility?  Na o kile wa itemogela hlaelelo ya diokobatsi tša madi ya magolo mo kliniking? | Yes/Ee 1  No/Aowa 2  N/A 3 | H10 |

| **CORE: HISTORY OF DIABETES / DIPOTŠITŠO TŠE BOHLOKWA: TŠA MABAPI LE BOLWETŠI BJA SWIKIRI** |
| --- |

| **NO** | **Question/*Po*tšišo** | **Answer/Karabo** | **Code** |
| --- | --- | --- | --- |
| 47 | Have you ever had your blood sugar measured by a doctor or other health worker?  A naa o kile wa lekolwa mabapi le bolwetši bja swikiri ke ngaka goba mošomedi o mongwe wa tša maphelo? | Yes/Ee 1  No/Aowa 2 | DM1 |
| 48 | Have you ever been told by a doctor or other health worker that you have raised blood sugar or diabetes?  A naa o kile wa botšwa ke ngaka goba mošomedi o mongwe wa tša maphelo gore kelo ya swikiri | Yes/Ee 1  No/Aowa 2 | DM2 |
| 49 | Do you have drugs (medication) that you have taken in the past two weeks?  Na o na diokobatši (dihlare) tšeo o di nwelego mo dibekeng tše pedi tša go feta. | Yes/Ee 1  No/Aowa 2  N/A 3 | DM3 |
| 50 | Do you have a special prescribed diet?  Dijo tšeo o di kgethetšwego ke ngaka ? | Yes/Ee 1  No/Aowa 2 | DM4 |
| 51 | Have you ever been advice to lose weight?  Na o eleditšwe goba go fokotša boima bja mmele? | Yes/Ee 1  No/Aowa 2 | DM5 |
| 52 | Have you received an advice or treatment to stop smoking?  Na o kile wa eletšwa goba newa kalafo ya go tlogela go kgoga motšoko? | Yes/Ee 1  No/Aowa 2  N/A 3 | DM6 |
| 53 | Have you received an advice to start or do more exercise?  Na o kile wa eletšwa ya go thoma go itšhidulla kudu? | Yes/Ee 1  No/Aowa 2 | DM7 |
| 54 | Any knowledge of behavioural risk factors for Diabetes?  A naa o tseba mekgwa yeo e bakago bolwetši bja swikiri? | Yes/Ee 1  No/Aowa 2 | DM8 |
| 55 | Have you ever experience shortage of DM treatment in the facility?  Na o kile wa itemogela hlaelelo ya diokobatsi mo kliniking? | Yes/Ee 1  No/Aowa 2  N/A 3 | DM9 |
| 56 | Is there a family history of diabetic mellitus?  Na go na le wa leloko yo a kilego a swarwa ke bolwetsi bja swikiri? | Yes/Ee 1  No/Aowa 2 | DM10 |
| 57 | Have you had hypertension & Diabetes campaign in the facility?  Na kliniki e dira fahlošo ka bolwetši bja swikiri le madiamagolo ka kakaretšo? | Yes/Ee 1  No/Aowa 2  Sometimes / e sego ka mehla 3 | DM11 |
| 58 | Does the facility provide health talks on hypertension & diabetes daily?  Na kliniki e ruta ka madiamago le swikiri ka mehla? | Yes/Ee 1  No/Aowa 2  Sometimes / e sego ka mehla 3 | DM12 |
| 59 | When last did the facility had a campaign regarding behavioural risk factors of hypertension & Diabetes?  Ke neng la bofelo kliniki e kilego ya fahloša setšhaba ka mekgwa yeo bakago bolwetši bja madiamagolo le swikiri? | A week ago/beke e fetilego 1  A month ago/ kgwedi e fetilego 2  6 month ago/ dikwedi tše tsela tse fetilego 3  12 month ago/ dikgwedi tse 12 tse fetilego 4  Never had one/ga se ya dirwa 5 | DM13 |
